# Additive effects of genetic variants associated with intraocular pressure in primary open-angle glaucoma

**DOI:** 10.1371/journal.pone.0183709

**Published:** 2017-08-23

**Authors:** Fumihiko Mabuchi, Nakako Mabuchi, Yoichi Sakurada, Seigo Yoneyama, Kenji Kashiwagi, Hiroyuki Iijima, Zentaro Yamagata, Mitsuko Takamoto, Makoto Aihara, Takeshi Iwata, Kazuhide Kawase, Yukihiro Shiga, Koji M. Nishiguchi, Toru Nakazawa, Mineo Ozaki, Makoto Araie, for the Japan Glaucoma Society Omics Group (JGS-OG)

**Affiliations:** 1 Department of Ophthalmology, Faculty of Medicine, University of Yamanashi, Yamanashi, Japan; 2 Department of Health Sciences, Faculty of Medicine, University of Yamanashi, Yamanashi, Japan; 3 Department of Ophthalmology, Tokyo Metropolitan Police Hospital, Tokyo, Japan; 4 Department of Ophthalmology, Graduate School of Medicine, University of Tokyo, Tokyo, Japan; 5 Division of Molecular and Cellular Biology, National Institute of Sensory Organs, National Hospital Organization Tokyo Medical Center, Tokyo, Japan; 6 Gifu University Hospital, Gifu, Japan; 7 Department of Ophthalmology, Tohoku University Graduate School of Medicine, Miyagi, Japan; 8 Department of Advanced Ophthalmic Medicine, Tohoku University Graduate School of Medicine, Miyagi, Japan; 9 Department of Retinal Disease Control, Tohoku University Graduate School of Medicine, Miyagi, Japan; 10 Ozaki Eye Hospital, Miyazaki, Japan; 11 Kanto Central Hospital of the Mutual Aid Association of Public School Teachers, Tokyo, Japan; Harvard Medical School, UNITED STATES

## Abstract

To investigate the association between the additive effects of genetic variants associated with intraocular pressure (IOP) and IOP, vertical cup-to-disc ratio (VCDR), and high tension glaucoma (HTG) or normal tension glaucoma (NTG) as phenotypic features of primary open-angle glaucoma (POAG), and to evaluate the clinical usefulness of the additive effects of IOP-related genetic variants for predicting IOP elevation, Japanese patients with HTG (n = 255) and NTG (n = 261) and 246 control subjects were genotyped for nine IOP-related genetic variants near *CAV2*, *GAS7*, *GLCCI1/ICA1*, *ABCA1*, *ARHGEF12*, *FAM125B*, *FNDC3B*, *ABO*, and *PTPRJ/AGBL2*. The total number of risk alleles of these genetic variants was calculated for each participant as a genetic risk score (GRS), and the association between the GRS and the maximum IOP, mean VCDR, and phenotype (HTG or NTG) of POAG was evaluated. As the GRS increased, the maximum IOP (P = 0.012) and VCDR (P = 0.010) significantly increased. The GRS (9.1±1.9) in patients with HTG was significantly higher (P = 0.011) than that (8.7±1.8) in control subjects. The patients with GRS≥12 as a cut-off value had a 2.54 times higher (P = 0.0085) risk on HTG (maximum IOP≥22mmHg) compared with all patients. The IOP-related GRS approach substantiated that the IOP and VCDR were increased by the additive effects of IOP-related genetic variants in POAG. The high IOP-related GRS in patients with HTG but not NTG shows that there are differences in the genetic background between HTG and NTG and supports the notion that the phenotype (HTG or NTG) in patients with POAG depends on the additive effects of IOP-related genetic variants. The above-mentioned cut-off value of IOP-related GRS may be clinically useful for predicting the risk of IOP elevation.

## Introduction

The optic nerve carries impulses for sight from the retina to the brain. It is composed of retinal nerve fibers that bundle together and exit to the brain through the optic disc. The optic disc has a center portion called the "cup", and retinal nerve fiber loss causes the cup to become larger in comparison to the optic disc. Glaucoma is an optic neuropathy with excavated optic disc cupping and progressive visual field loss, and vertical elongation of the optic disc cupping is a characteristic feature. The vertical cup-to-disc ratio (VCDR) is taken to be the longest vertical cup diameter divided by the longest vertical disc diameter, and is a parameter commonly used in the clinical management of glaucoma. An increased VCDR indicates the occurrence of glaucomatous changes of the optic disc.[1,2] Although the elevation of intraocular pressure (IOP) is recognized as a most important risk factor for glaucoma,[3] the pathogenesis and etiology of IOP elevation in patients with glaucoma is still not well understood. Primary open-angle glaucoma (POAG), the most prevalent form of glaucoma, is clinically classified into high tension glaucoma (HTG), in which an elevated IOP is observed, and normal tension glaucoma (NTG), in which the IOP is consistently within the statistically normal range for the population. A family history of glaucoma is also a major clinical risk factor for POAG,[4–9] and it was reported that a family history of glaucoma reflected the influence of genetic variants that predispose individuals to POAG.[10] In addition, the heritability of IOP has been reported.[11–18] Therefore, the investigation of genes that influence the IOP may help elucidate the differences in the genetic background between HTG and NTG as well as the genetic mechanisms of IOP regulation. Genetic variants including a predisposition to POAG can be classified into IOP-related and non-IOP-related variants, the latter of which includes other factors associated with vulnerability of the retinal ganglion cell (RGC) and/or optic nerve independent of IOP. Recent genome-wide association studies (GWASs) have identified several IOP-related genetic variants, [19–27] and the additive effects of IOP-related genetic variants on POAG (combined HTG and NTG) have been reported.[28] However, the additive effects of IOP-related genetic variants on the IOP or VCDR are unknown, and while it is presumed that the additive effects of IOP-related genetic variants would predominate in patients with HTG rather than NTG, this has yet to be confirmed. We therefore investigated the association between the additive effects of IOP-related genetic variants and IOP itself, VCDR as a clinical parameter of glaucoma, and HTG or NTG as phenotypic features of POAG. Furthermore, we also examined the clinical usefulness of the additive effects of IOP-related genetic variants in improving glaucoma risk (IOP elevation) prediction.

## Materials and methods

### Subjects

Japanese patients with POAG were recruited from the ophthalmology practices at the Enzan Municipal Hospital, Oizumi Clinic, Uenohara City Hospital, and Yamanashi University Hospital in Yamanashi Prefectures, Japan. POAG was diagnosed when an open anterior chamber angle was detected on a gonioscopic examination and the glaucomatous visual field defect (nasal step and/or partial arcuate visual field defect) with compatible optic disc cupping (thinning of the optic disc rim and/or enlargement of the optic disc cupping) was observed on automated static perimetry (Humphrey Visual Field Analyzer 30–2: HFA30-2, Humphrey Instruments, San Leandro, CA, USA). A visual field defined by false-positive results, false-negative results, or fixation losses not exceeding 33% was used as reliable. Some patients had 1 measurement of visual field test at the time of blood sampling, although most patients had reproducible visual field defects. In addition, patients were diagnosed with HTG when they had at least 1 previous measurement of IOP≥22mmHg with a Goldmann applanation tonometer. Patients with NTG showed an IOP≤21mmHg each time they were tested. Twenty-four-hour IOP variation was measured in the sitting position for 53.6% of NTG patients. Patients who had a history of eye surgery, including laser treatment, before the diagnosis of POAG were excluded from the present study. The control subjects, who were recruited from participating institutions, included Japanese individuals who were over 40 years of age, with an IOP≤21mmHg, who exhibited no glaucomatous cupping of the optic disc (no thinning of disc rim and VCDR≤0.4), and who had no family history of glaucoma. Comprehensive ophthalmologic examinations including both slit-lamp biomicroscopy and fundoscopy were performed for all participants. The highest IOP in both eyes chosen from all measured IOPs on medical records was considered as the maximum IOP. IOPs measured after surgical treatments were excluded. The VCDR was determined by a glaucoma specialist (F.M) using a 90-diopter lens at the time of blood sampling, and the mean VCDR in both eyes was used for analyses. The study protocol was prospectively approved by the Ethics Committee of University of Yamanashi and written informed consent was obtained from all study participants. The present study was conducted in accordance with the Declaration of Helsinki.

### Genomic DNA genotyping

Genomic DNA was purified from peripheral blood with a Flexi Gene® DNA Kit (QIAGEN, Valencia, CA, USA). Nine genetic variants identified as IOP-related genetic variants on GWAS, including rs1052990 (near gene; caveolin 2: *CAV2*),[25,29] rs11656696 (growth arrest specific 7: *GAS7*),[21] rs59072263 (glucocorticoid induced 1/islet cell autoantigen 1: *GLCCI1/ICA1*),[22] rs2472493 (ATP binding cassette subfamily A member 1: *ABCA1*),[23–25] rs58073046 (Rho guanine nucleotide exchange factor 12: *ARHGEF12*),[27] rs2286885 (family with sequence similarity 125, member B: *FAM125B*),[26] rs6445055 (fibronectin type III domain containing 3B: *FNDC3B*),[25] rs8176743 (ABO blood group: *ABO*),[25] and rs747782 (protein tyrosine phosphatase, receptor type J: *PTPRJ*),[25] were genotyped using TaqMan single nucleotide polymorphism genotyping assays (Applied Biosystems [ABI], Foster City, CA, USA). Assays were performed on a 7300/7500 Real-Time PCR System (ABI, Foster City, CA, USA) according to the manufacturer’s instructions. The IOP-related genetic variants on chromosome 2p found in Afro-Caribbean[19], African-American[30], and Indian[31] populations were not included in the present study, because no significant associations between these genetic variants and POAG have been observed in the Japanese population.[32] Similarly, the IOP-related genetic variants near the transmembrane and coiled-coiled domains 1 (*TMCO1*) gene identified in Caucasian[20,21], Pakistani,[33] and Chinese[34] populations were not also included because these genetic variants were not polymorphic in the Japanese population.

### Statistical analysis

Data analysis was performed using SAS statistical software (SAS Institute Inc., Cary, NC, USA). A multiple linear regression analysis was carried out with the maximum IOP as a dependent variable and age, gender, and the risk alleles of the 9 IOP-related genetic variants as independent variables to evaluate the effects of individual IOP-related genetic variants for the IOP. To evaluate the additive effects of IOP-related genetic variants, the total number of risk alleles (range: 0 to 18) of the 9 IOP-related genetic variants was calculated for each participant as a genetic risk score (GRS). To assess the association between this IOP-related GRS and the IOP or VCDR, a multiple linear regression analysis was carried out with the maximum IOP or the mean VCDR as dependent variables and age, gender, and the GRS as independent variables. From a clinical diagnostic point of view, the GRS was compared among the control subjects and patients with NTG and HTG using an analysis of variance (ANOVA) followed by Bonferroni post hoc test. A logistic regression analysis was carried out to study the effects of the GRS compared between the control subjects and patients with NTG or HTG, adjusted for age and gender. The ratio of patients with HTG (maximum IOP≥22mmHg) to the control subjects (maximum IOP≤21mmHg) was compared with respect to the GRS using a Chi-square test. A value of P<0.05 was considered to be statistically significant without a value of P<0.016 (0.05/3) for Bonferroni post hoc test.

## Results

Seven hundred and sixty-two Japanese patients, including 516 patients with POAG (255 patients with HTG and 261 patients with NTG) and 246 control subjects, were enrolled in the present study. The demographic and clinical data for all participants are shown in Table 1. The mean age at the time of blood sampling was 63.8±13.8 years (standard deviation) in patients with POAG and 67.7±11.2 years in the control subjects.

**Table 1:** Demographic and clinical data in patients with POAG and control subjects.

	Control (n = 246)	POAG	
		NTG (n = 261)	HTG (n = 255)
Age at blood sampling (years)	67.7 ± 11.2	63.8 ± 13.3	63.7 ± 14.2
Age at diagnosis (years)	-	57.6 ± 13.0	55.6 ± 15.0
Male gender, n (%)	90 (36.6)	102 (39.1)	156 (61.2)
Refractive error (diopter)	-0.2 ± 2.0	-2.0 ± 3.4	-2.1 ± 3.2
Maximum intraocular pressure (mmHg)	15.0 ± 2.6	18.4 ± 1.9	28.6 ± 8.3
Mean vertical cup-to-disc ratio in both eyes	0.34 ± 0.08	0.83 ± 0.10	0.85 ± 0.13
Familial history of glaucoma, n (%)	0 (0)	60 (23.0)	74 (29.0)

POAG: primary open-angle glaucoma, NTG: normal tension glaucoma, HTG: high tension glaucoma, Continuous variables are expressed as mean ± standard deviation.

The mean of maximum IOP was 23.4±7.9mmHg in patients with POAG (18.4±1.9mmHg and 28.6±8.3mmHg in patients with NTG and HTG respectively) and 15.0±2.6mmHg in the control subjects. The result of a multiple linear regression analysis with the maximum IOP as a dependent variable and age, gender, and the risk alleles of the 9 IOP-related genetic variants as independent variables was shown in Table 2.

**Table 2:** Result of a multiple linear regression analysis using the maximum IOP as a dependent variable.

Independent variables (Near gene)	Beta	SE	P value
Age	-0.077	0.021	0.031
Male gender	0.20	0.55	< 0.0001
Risk alleles of IOP-related genetic variants for IOP elevation			
rs1052990 T allele (*CAV2*)	0.080	0.48	0.025
rs11656696 C allele (*GAS7*)	-0.004	0.40	0.90
rs59072263 G allele (*GLCCI1/ICA1*)	0.041	0.69	0.26
rs2472493 C allele (*ABCA1*)	-0.008	0.39	0.83
rs58073046 G allele (ARHGEF12)	0.041	0.46	0.25
rs2286885 T allele (*FAM125B*)	0.045	0.41	0.21
rs6445055 G allele (*FNDC3B*)	0.008	0.44	0.82
rs8176743 A allele (*ABO*)	0.078	0.57	0.027
rs747782 G allele (*PTPRJ*)	0.031	0.40	0.38

IOP: intraocular pressure, Beta: standardized regression coefficient, SE: standard error, *CAV2*: caveolin 2, *GAS7*: growth arrest specific 7, *GLCCI1/ICA1*: glucocorticoid induced 1/islet cell autoantigen 1, *ABCA1*: ATP binding cassette subfamily A member 1, *ARHGEF12*: Rho guanine nucleotide exchange factor 12, *FAM125B*: family with sequence similarity 125, member B, *FNDC3B*: fibronectin type III domain containing 3B, *ABO*: ABO blood group, *PTPRJ*: protein tyrosine phosphatase, receptor type J. F change = 5.0, P < 0.0001.

### Association between the GRS of multi-locus IOP-related genetic variants and the IOP or VCDR

The results of multiple linear regression analysis with the maximum IOP or the mean VCDR as dependent variables and age, gender, and the GRS as independent variables were shown in Tables 3 and 4. There was a significant relationship (Beta = 0.090, P = 0.012, Table 3) between the GRS and the maximum IOP, and as the GRS increased, so did the maximum IOP. Similarly, a significant relationship (Beta = 0.093, P = 0.010, Table 4) was noted between the GRS and the mean VCDR, and as the GRS increased, so did the VCDR.

**Table 3:** Result of multiple linear regression analysis using the maximum IOP as a dependent variable.

Age	-0.079	0.021	0.027
Male gender	0.20	0.55	< 0.0001
GRS (Total number of risk alleles of 9 IOP-related genetic variants)	0.090	0.15	0.012

IOP: intraocular pressure, Beta: standardized regression coefficient, SE: standard error, GRS: genetic risk score, F change = 15.8, P < 0.0001.

**Table 4:** Result of multiple linear regression analysis using the VCDR as a dependent variable.

Independent variables	Beta	SE	P value
Age	-0.059	0.001	0.10
Male gender	0.11	0.019	0.0019
GRS (Total number of risk alleles of 9 IOP-related genetic variants)	0.093	0.005	0.010

VCDR: vertical cup-to-disc ratio, Beta: standardized regression coefficient, SE: standard error, GRS: genetic risk score, F change = 7.2, P < 0.0001.

### Association between the GRS of multi-locus IOP-related genetic variants and HTG or NTG as phenotypic features of POAG

**Fig 1:**
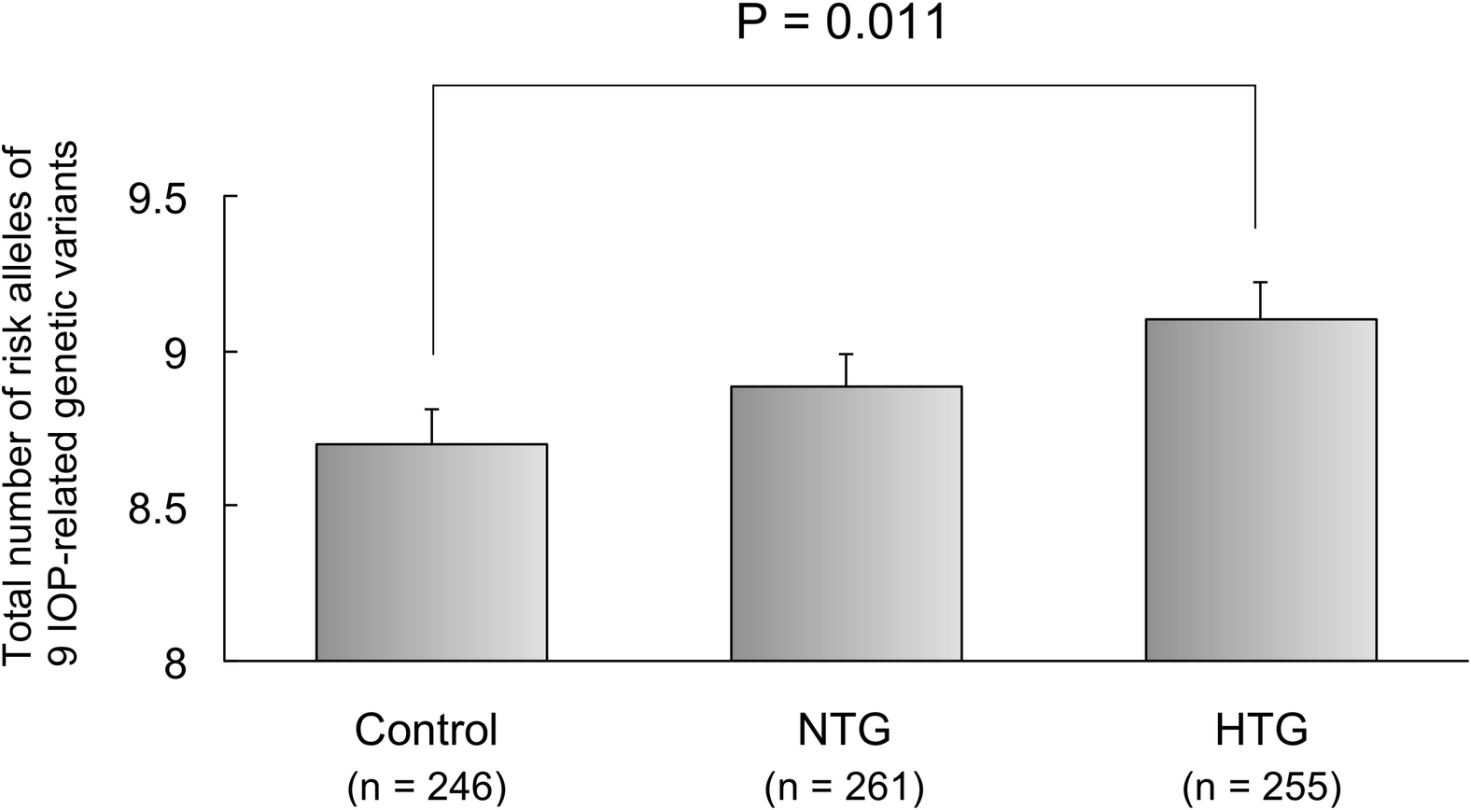
Association between the phenotype (HTG or NTG) of primary open-angle glaucoma and the additive effects of multi-locus IOP-related genetic variants. The IOP-related genetic risk score calculated as the total number of risk alleles of the 9 IOP-related genetic variants in patients with HTG was significantly higher (P = 0.040, analysis of variance followed by Bonferroni post hoc test) than that in the control subjects. IOP: intraocular pressure, HTG: high tension glaucoma, NTG: normal tension glaucoma.

**Fig 2:**
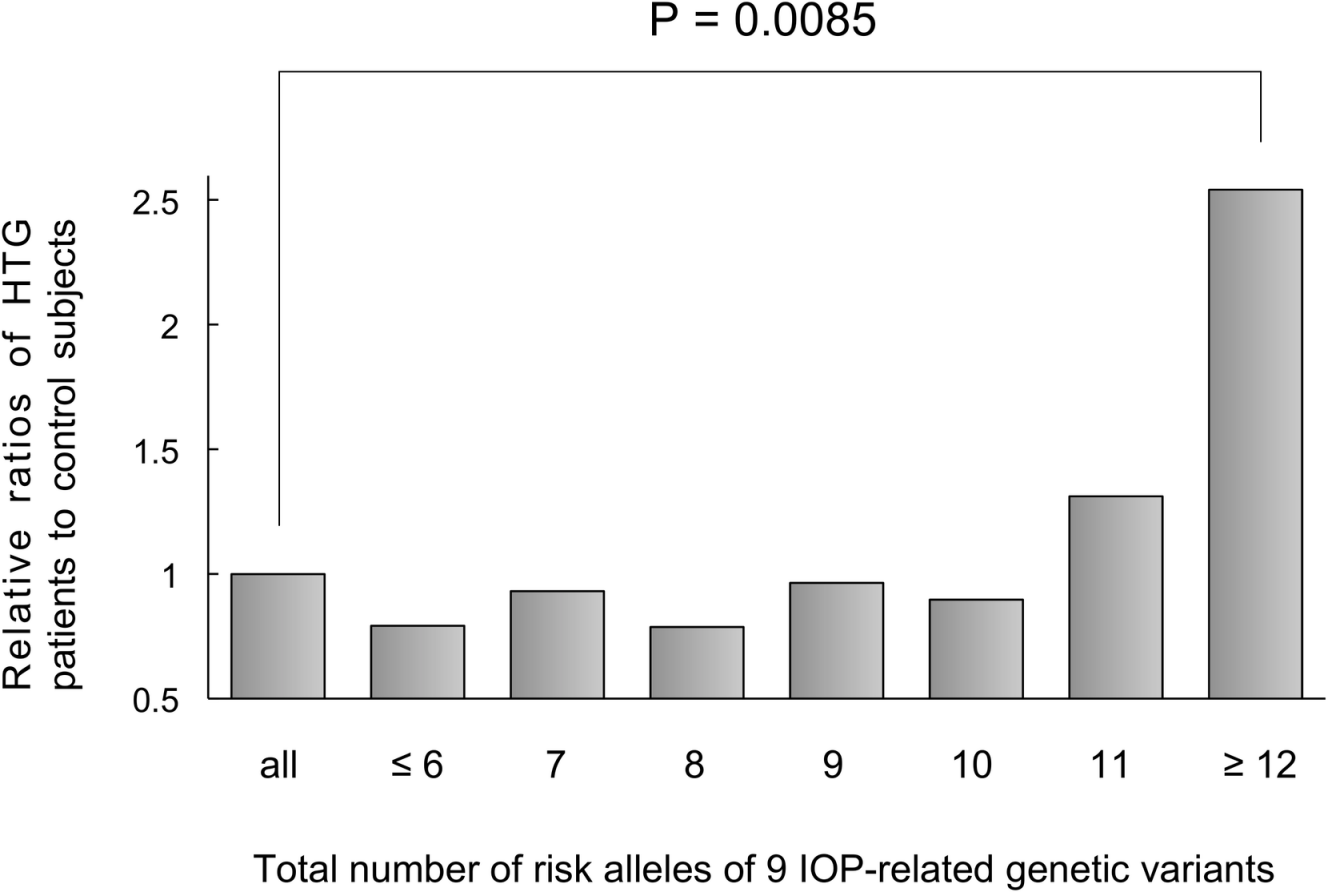
Association between the IOP-related GRS and the relative ratio of HTG patients to control subjects. When the ratio (255 HTG patients/246 control subjects) of all HTG patients (maximum IOP≥22mmHg) to all control subjects (maximum IOP≤21mmHg) was set as 1, the ratio (29 HTG patients/11 control subjects) of HTG to control in patients with the IOP-related GRS calculated as the total number of risk alleles of the 9 IOP-related genetic variants ≥12 was 2.54, which was significantly larger (Chi-square test) than that in all patients. IOP: intraocular pressure, GRS: genetic risk score, HTG: high tension glaucoma.

The GRS (9.1±1.9) in patients with HTG was significantly higher (P = 0.040, ANOVA; P = 0.011, Bonferroni post hoc test) than that (8.7±1.8) in the control subjects. (Fig 1) After adjusting for age and gender, almost 1.1-fold increased risk of HTG was found for the GRS (P = 0.030, odds ratio [OR]: 1.12 per risk allele, 95% confidence interval: 1.01 to 1.24, Table 5). The ratio (29 HTG patients/11 control subjects) of HTG (maximum IOP≥22mmHg) patients to control (maximum IOP≤21mmHg) subjects in patients with GRS≥12 was 2.54 times larger (P = 0.0085, Chi-square test) than that (255 HTG patients/246 control subjects) in all patients. (Fig 2)

**Table 5:** Results of logistic regression analysis between NTG or HTG patients and control subjects.

Variables	NTG		HTG	
	P value	Odds Ratio (95% CI)	P value	Odds Ratio (95% CI)
Age (per year)	0.0009	0.98 (0.96 to 0.99)	0.012	0.98 (0.97 to 0.99)
Male gender	0.70	1.07 (0.75 to 1.55)	< 0.0001	2.63 (1.82 to 3.80)
GRS (Total number of risk alleles of 9 IOP-related genetic variants)	0.41	1.05 (0.94 to 1.16)	0.030	1.12 (1.01 to 1.24)

NTG: normal tension glaucoma, HTG: high tension glaucoma, CI: confidence Interval, GRS: genetic risk score, IOP: intraocular pressure.

## Discussion

We showed a significant relationship between the IOP and the IOP-related GRS calculated as the total number of risk alleles of the 9 IOP-related genetic variants, indicating that the greater the number of IOP-related genetic variants, the higher the IOP in patients with POAG. To our knowledge, this is the first report to substantiate that the IOP is elevated by the additive effects of IOP-related genetic variants. The IOP-related GRS was associated more strongly with the IOP compared with the evaluations of individual IOP-related genetic variants. The stronger effect of the IOP-related GRS supports the notion that the IOP is regulated by multiple genes. To evaluate the additive effects of IOP-related genetic variants, the total number of risk alleles of multi-locus IOP-related genetic variants was used as an unweighted GRS in the present study. Given that the unweighted GRS approach assumed that all risk alleles had the same magnitude of effect on the IOP, the results might not precisely reflect the additive effects of IOP-related genetic variants. Therefore, in the previous study reported the additive effects of IOP-related genetic variants on POAG,[28] a logistic regression model was used to estimate the risk (OR) of glaucoma for each risk allele of the IOP-related genetic variants, and the sum of the logarithmically converted ORs of multi-locus IOP-related genetic variants was used as a weighted GRS. In the present study, the results by the same weighted GRS approach (See S1–S3 Tables and S1 Fig) were fundamentally the same as those by the unweighted GRS approach.

As for the association between the VCDR and the additive effects of IOP-related genetic variants, there was a significant relationship between the VCDR and the IOP-related GRS, indicating that the VCDR in patients with POAG is enlarged, as the number of IOP-related genetic variants increases. Previous GWASs have identified 18 genetic loci associated with VCDR,[35–37] and Tham et al.[28] reported that a higher GRS of these 18 VCDR-related genetic loci was associated with an increased risk of POAG. These genetic loci appear to contribute to glaucomatous optic neuropathy (enlargement of the VCDR) in patients with POAG as a non-IOP-related genetic variant (locus) associated with the vulnerability of RGC/optic nerve, because a significant association between these genetic loci and the IOP has not been identified. In fact, the IOP-related genetic variants are not included in these VCDR-related genetic loci. However, our findings show that the IOP-related genetic variants, when aggregated together, have a significant association with VCDR, although the IOP-related genetic variants may have relatively small effects on VCDR individually. This result is due to the greater statistical power conferred by the GRS approach than individual genotype analyses and supports the notion that POAG is a complex genetic disorder induced by multi-locus IOP-related genetic variants as well as non-IOP-related genetic variants. The correlation between the IOP-related GRS and VCDR is reasonable, since the most important risk factor for enlargement of the VCDR used as a clinical parameter for the occurrence and/or progression of POAG is an elevation of IOP regulated by the additive effects of IOP-related genetic variants. In fact, there was a significant correlation (Beta = 0.48, P<0.0001) between the maximum IOP and VCDR in the present study.

Tham et al.[28] also reported that a higher GRS derived from the 7 IOP-related genetic variants near *TMCO1*, *CAV2*, *GAS7*, *ABCA1*, *FNDC3B*, *ABO*, and *PTPRJ/AGBL2* (ATP/GTP binding protein like 2) was significantly associated with a greater risk of POAG. The POAG patients in their study were thought to include patients with HTG and NTG, because their study design was population-based and the IOP in their patients with POAG was 17.0±4.4mmHg (mean ± standard deviation).[28] The present study revealed a high IOP-related GRS in patients with HTG but not NTG, and a logistic regression analysis confirmed a significant relationship between the IOP-related GRS and HTG. These results are reasonable, as the IOP in patients with NTG is consistently within the statistically normal population range, indicating that the genetic background differs between patients with HTG and NTG. We previously reported that the GRS of multi-locus non-IOP-related genetic variants in patients with HTG as well as NTG was significantly higher than that in control subjects,[10] indicating that the vulnerability of the RGC/optic nerve due to non-IOP-related genetic variants also plays an important role in the pathogenesis of HTG, in addition to IOP elevation induced by IOP-related genetic variants. Based on those results, we proposed the following genetic mechanism of POAG.[10] Patients with a high non-IOP-related and low IOP-related GRS are expected to present with phenotypic features of NTG because these patients develop glaucomatous optic neuropathy by the vulnerability of the optic nerve due to non-IOP-related genetic variants without IOP elevation induced by IOP-related genetic variants. In contrast, those with a low non-IOP-related and high IOP-related GRS are expected to present with phenotypic features of ocular hypertension (OH), which means that there are no signs of glaucomatous optic neuropathy, although the IOP is elevated. Those with high non-IOP-related and IOP-related GRS are expected to present with phenotypic features of HTG because these patients develop glaucomatous optic neuropathy by the vulnerability of the optic nerve due to non-IOP-related genetic variants in addition to IOP elevation induced by IOP-related genetic variants. Our present finding of high IOP-related GRS in patients with HTG but not NTG supports this hypothesis, and the phenotype (HTG or NTG) in patients with POAG is presumed to depend on the IOP-related GRS. Scheetz et al.[38] reported that non-Hispanic white OH patients with *TMCO1* risk alleles participating in the Ocular Hypertension Treatment Study had a 12% higher cumulative frequency of glaucoma developing than OH patients with no *TMCO1* risk alleles, suggesting a difference in the genetic background between the patients with OH and HTG, although the *TMCO1* genetic variants are not non-IOP-related but IOP-related genetic variants. A genetic analysis via the GRS approach including an OH cohort would provide further insights into the complex genetic mechanisms of POAG.

Establishing the optimum method for evaluating POAG onset risk would be clinically useful for the early diagnosis and treatment of POAG. Because of the polygenic nature of glaucoma, the GRS approach may provide more conclusive insights on the clinical usefulness of genetic variants in POAG risk prediction than other approaches. Although one report found no clear evidence supporting the utility of the IOP-related GRS approach,[39] it was reported that participants in the top GRS tertile of the 7 multi-locus IOP-related genetic variants were 2.50 times more likely to have POAG than those in the bottom tertile.[28] Similar results using the multi-locus GRS derived from a few mutations causing POAG and/or non-IOP-related (VCDR-related) genetic variants have been reported.[28,37,39,40] In the present study, the ratio of HTG (maximum IOP≥22mmHg) patients to control (maximum IOP≤21mmHg) subjects in patients with the unweighted IOP-related GRS (total number of risk alleles of the 9 IOP-related genetic variants) ≥12 was almost 2.5 times larger than that in all patients as shown in Fig 2, indicating that the patients with this GRS are at a high risk for IOP elevation beyond the normal population range and should be followed up carefully. This cut-off value for the IOP-related GRS may be clinically useful for predicting risk of IOP elevation, at least in the Japanese population, although further verification with different cohorts of HTG patients and control subjects is required. Concretely, using this cut-off value, it may be possible to predict the probability of future IOP elevation (IOP≥22mmHg) for individuals who have high POAG risks such as family history of severe glaucoma and/or early-onset glaucoma suspects. This cut-off value may also provide clinicians with useful information for deciding whether or not active treatments should be performed in individuals with preperimetric glaucoma. However, the prevalence of HTG is lower in the Japanese population than in other ethnicities (only 8% of Japanese patients with POAG have HTG).[41] Although IOP-related genetic variants on chromosome 2p have been found in Afro-Caribbean[19], African-American[30], and Indian[31] populations, no significant associations between these genetic variants and POAG (NTG and HTG) have been observed in the Japanese population.[32] Furthermore, the IOP-related genetic variants near the *TMCO1* gene identified in Caucasian,[20,21] Pakistani,[33] and Chinese[34] populations are not polymorphic in the Japanese population. Since the genetic background differs between ethnicities, further studies should be performed to verify whether the cut-off value of IOP-related GRS described in the present study is useful for predicting IOP elevation in other ethnic populations.

As the limitations of the present study, there is a certain limit to an IOP evaluation. In the previous study with IOP-related GRS approach,[28] they adjusted the IOP by adding 25% to the measured IOP level for eyes receiving IOP lowering medication. In the present study, we evaluated the maximum IOP but not the adjusted IOP because the effect, number, and kind of IOP lowering medicines (eye drops) differed by patients. In addition, the IOP was not also adjusted according to the central corneal thickness. The VCDR was estimated using a 90-diopter lens and not actually measured with a fundus camera or an optical coherence tomography. More significant P values might be obtained by IOP adjustment according to the IOP lowering medication and/or central corneal thickness and VCDR evaluation using image analyzing devices. The previous study with IOP-related GRS approach[28] was a population-based case-control study. However, ours was a clinic-based case-control study which may have introduced selection bias. Since the participants of the present study consist of Japanese subjects only, further verification for the generalization of our findings may be required in the other ethnic populations.

In conclusion, the IOP-related GRS approach substantiated that the IOP and VCDR were elevated and enlarged respectively by the additive effects of IOP-related genetic variants in POAG. The high IOP-related GRS in patients with HTG but not NTG showed that there are differences in the genetic background between HTG and NTG and supports the notion that the phenotype (HTG or NTG) in patients with POAG depends on the additive effects of IOP-related genetic variants. The cut-off value of IOP-related GRS described above may be clinically useful for predicting the risk of IOP elevation.

## Supporting information

S1 Table Result of multiple linear regression analysis using the maximum IOP as a dependent variable. (DOC)

S2 Table Result of multiple linear regression analysis using the VCDR as a dependent variable. (DOC)

S3 Table Results of logistic regression analysis between NTG or HTG patients and control subjects. (DOC)

S1 Fig Association between the phenotype (HTG or NTG) of primary open-angle glaucoma and the additive effects of multi-locus IOP-related genetic variants. The risk (odds ratio) of IOP elevation (IOP≥22mmHg) for each risk allele of 9 IOP-related genetic variants was calculated using a logistic regression analysis, and the sum of the logarithmically converted odds ratios of these variants was used as a weighted IOP-related GRS. The weighted IOP-related GRS in patients with HTG was significantly higher (P = 0.011, analysis of variance followed by Bonferroni post hoc test) than that in the control subjects. IOP: intraocular pressure, GRS: genetic risk score, HTG: high tension glaucoma, NTG: normal tension glaucoma.
(PDF)
