# Supplementary figures and images for: Additive effects of genetic variants associated with intraocular pressure in primary open-angle glaucoma
Source: PLoS One. 2017 Aug 23;12(8):e0183709. doi: 10.1371/journal.pone.0183709 (PMC5568337; doi:10.1371/journal.pone.0183709)

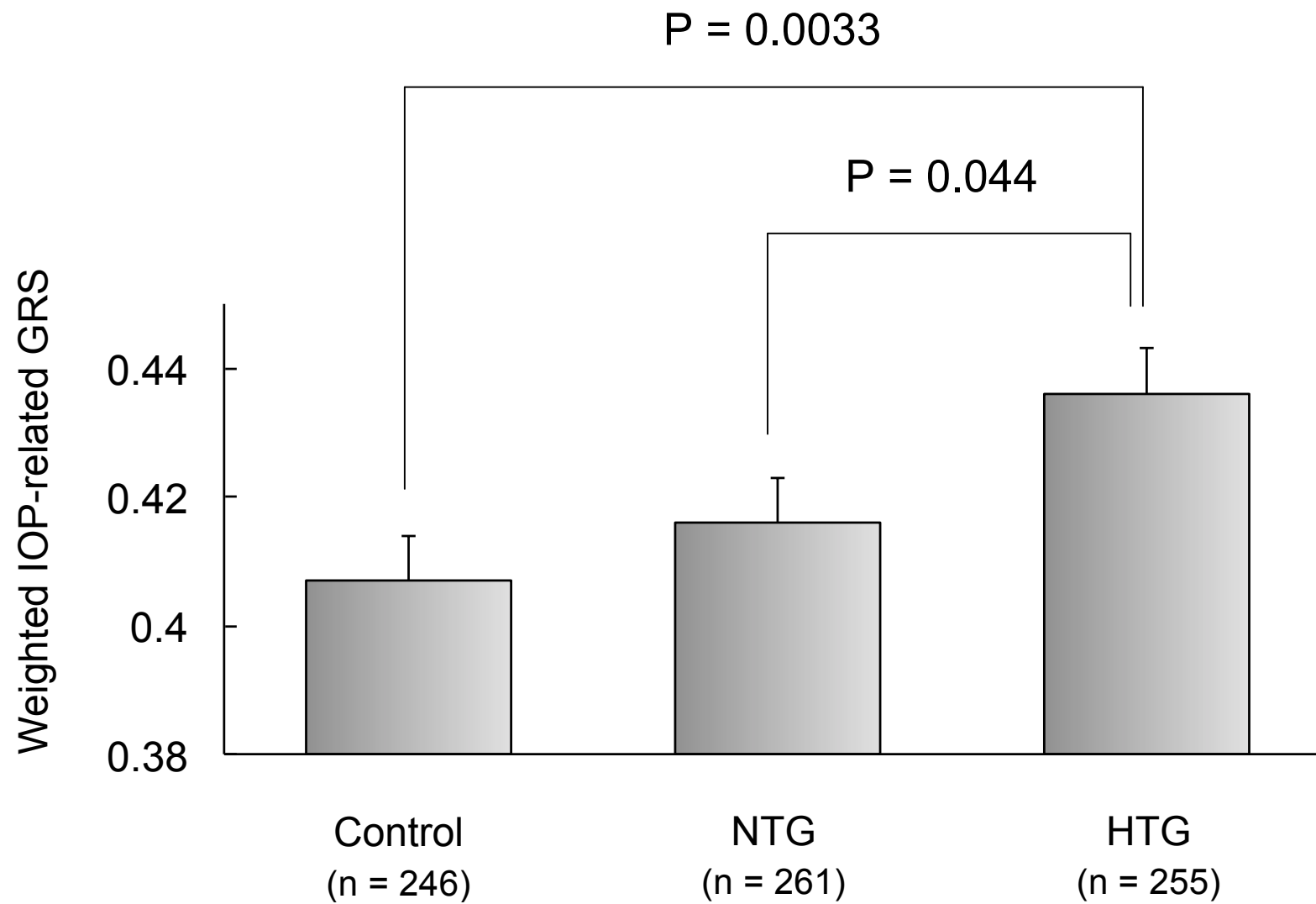

Supplement: S1 Fig — The risk (odds ratio) of IOP elevation (IOP≥22mmHg) for each risk allele of 9 IOP-related genetic variants was calculated using a logistic regression analysis, and the sum of the logarithmically converted odds ratios of these variants was used as a weighted IOP-related GRS. The weighted IOP-related GRS in patients with HTG was significantly higher (P = 0.011, analysis of variance followed by Bonferroni post hoc test) than that in the control subjects. IOP: intraocular pressure, GRS: genetic risk score, HTG: high tension glaucoma, NTG: normal tension glaucoma. (PDF) [file pone.0183709.s004.pdf]
