# Supplementary material for: Additive effects of genetic variants associated with intraocular pressure in primary open-angle glaucoma
Source: PLoS One. 2017 Aug 23;12(8):e0183709. doi: 10.1371/journal.pone.0183709 (PMC5568337; doi:10.1371/journal.pone.0183709)
Supplement: Supplementary file 2 [file pone.0183709.s001.doc]

S1 Table. Result of multiple linear regression analysis using the maximum IOP as a dependent variable

Independent variables	Beta	SE	P value
Age (years)	-0.080	0.021	0.025
Male gender	0.20	0.55	< 0.0001
Weighted IOP-related GRS*	0.11	2.42	0.0022

IOP: intraocular pressure, Beta: standardized regression coefficient, SE: standard error, GRS: genetic risk score.
*The risk (odds ratio) of IOP elevation (IOP ≥ 22mmHg) for each risk allele of 9 IOP-related genetic variants was calculated using a logistic regression analysis, and the sum of the logarithmically converted odds ratio of these variants was used as a weighted IOP-related GRS. F change = 16.9, P < 0.0001.
