# Supplementary material for: Additive effects of genetic variants associated with intraocular pressure in primary open-angle glaucoma
Source: PLoS One. 2017 Aug 23;12(8):e0183709. doi: 10.1371/journal.pone.0183709 (PMC5568337; doi:10.1371/journal.pone.0183709)
Supplement: Supplementary file 3 [file pone.0183709.s002.doc]

S2 Table. Result of multiple linear regression analysis using the VCDR as a dependent variable

Independent variables	Beta	SE	P value
Age (years)	-0.062	0.001	0.084
Male gender	0.11	0.019	0.0017
Weighted IOP-related GRS*	0.084	0.082	0.019

VCDR: vertical cup-to-disc ratio, Beta: standardized regression coefficient, SE: standard error, IOP: intraocular pressure, GRS: genetic risk score. *The risk (odds ratio) of IOP elevation (IOP ≥ 22mmHg) for each risk allele of 9 IOP-related genetic variants was calculated using a logistic regression analysis, and the sum of the logarithmically converted odds ratios of these variants was used as a weighted IOP-related GRS. F change = 6.8, P = 0.0002.
