# Supplementary material for: Additive effects of genetic variants associated with intraocular pressure in primary open-angle glaucoma
Source: PLoS One. 2017 Aug 23;12(8):e0183709. doi: 10.1371/journal.pone.0183709 (PMC5568337; doi:10.1371/journal.pone.0183709)
Supplement: Supplementary file 4 [file pone.0183709.s003.doc]

S3 Table. Results of logistic regression analysis between NTG or HTG patients and control subjects

Variables	NTG		HTG	
	P value	Odds ratio (95% CI)	P value	Odds ratio (95% CI)
Age (per year)	0.0008	0.98 (0.96 to 0.99)	0.012	0.98 (0.97 to 0.99)
Male gender	0.68	1.08 (0.75 to 1.55)	< 0.0001	2.67 (1.85 to 3.87)
Weighted IOP-related GRS* (per 0.1)	0.48	1.06 (0.90 to 1.25)	0.0057	1.26 (1.07 to 1.48)

NTG: normal tension glaucoma, HTG: high tension glaucoma, CI: confidence Interval, IOP: intraocular pressure, GRS: genetic risk score. *The risk (odds ratio) of IOP elevation (IOP≥22mmHg) for each risk allele of 9 IOP-related genetic variants was calculated using a logistic regression analysis, and the sum of the logarithmically converted odds ratios of these variants was used as a weighted IOP-related GRS.
